# SynFit: Synergistic Contrastive Learning for Multi-Objective Protein Fitness Prediction and Optimization

**DOI:** 10.64898/2026.05.21.726972

**Published:** 2026-05-26

**Authors:** Tony Tu, Wei Huang, Ziang Li, Kerr Ding, Yang Yang, Yunan Luo

**Affiliations:** 1 School of Computational Science and Engineering, Georgia Institute of Technology; 2 Department of Chemistry and Biochemistry, University of California, Santa Barbara; 3 Department of Bioengineering, University of California, Santa Barbara; 4 Howard Hughes Medical Institute, University of California Santa Barbara

## Abstract

Proteins function through a complex interplay of structural and biochemical properties, and mutations can reshape these properties to generate fitness landscapes spanning multiple functional objectives. A central challenge in protein engineering is the need to simultaneously optimize multiple properties. In biocatalysis, for example, practical enzyme development routinely requires the concurrent optimization of catalytic activity, selectivity, stability, and substrate generality. However, despite recent advances in computational protein design and fitness prediction, most existing approaches treat these properties independently and do not explicitly capture the dependencies and trade-offs that govern real-world protein performance. We present SynFit, a multi-objective learning framework that integrates pretrained protein language models with experimental fitness measurements for protein fitness prediction and engineering. SynFit learns both shared and property-specific protein sequence representations through a synergistic contrastive learning strategy, enabling the identification of variants that simultaneously optimize multiple functional properties. Across a large-scale multi-fitness deep mutational scanning benchmark, SynFit consistently outperforms state-of-the-art supervised models trained on individual objectives and more accurately identifies variants that balance competing functional constraints. We further applied SynFit to multi-objective enzyme design for a new-to-nature biocatalytic enantioselective borylation reaction, providing a diverse array of novel cytochrome c sextuple variants in a single round of design with simultaneously improved catalytic activity and enantioselectivity that rival the best variants obtained through directed evolution. Together, these results establish SynFit as a general framework for multidimensional protein fitness prediction and highlight its potential to enable efficient multi-objective optimization in protein engineering, particularly in biocatalysis.

## 1 Introduction

Predicting the functional consequences of amino acid substitutions remains a fundamental challenge in protein science and engineering. Traditional computational approaches, including physics-based energy models and supervised machine learning (ML) methods, have provided important foundations for mutational effect prediction, but they often rely on engineered features, assay-specific supervision, and sufficiently large labeled datasets^1–7^. More recently, pretrained protein language models (pLMs) have substantially expanded the scope of sequence-to-fitness prediction by learning evolutionary and structural constraints from large protein sequence databases, enabling zero-shot predictions across diverse proteins and functions^8–15^. However, because these models primarily capture patterns of natural sequence variation rather than task-specific experimental readouts, their zero-shot predictions generally remain less accurate than models adapted to function-specific measurements^16–21^. In this context, integrating pretrained representations with limited experimental data through fine-tuning represents a highly appealing approach to bridging general sequence knowledge with function-specific prediction^17;21–26^.

Despite recent progress, most existing approaches remain focused on single-property prediction, whereas protein function is inherently multi-objective. In practice, successful protein engineering requires the simultaneous optimization of multiple functional properties. For example, in the field of biocatalysis and enzyme engineering, the development of useful biocatalysts often requires concurrent optimization of catalytic activity, selectivity, stability, and substrate generality. More broadly, mutations frequently affect stability, catalytic efficiency, binding affinity, expression, and selectivity in coupled and context-dependent ways^27–31^. These interdependencies give rise to complex fitness landscapes that cannot be readily decomposed into independent objectives. This problem is further exacerbated by the small and heterogeneous nature of experimental datasets, particularly in the low-data regime where models must disentangle shared sequence features from property-specific determinants. Collectively, these challenges highlight the need for methods that integrate pretrained sequence knowledge with limited experimental data while explicitly capturing dependencies and trade-offs across multiple functional properties.

Here, we introduce SynFit (synergistic contrastive learning for fitness prediction), a deep learning framework that integrates pretrained pLMs with experimental fitness measurements for multi-objective protein fitness prediction and engineering. SynFit combines a shared module that learns sequence features informative across diverse functions and a set of property-specific predictors that exploit the learned shared features for accurate multi-objective fitness prediction. To address the challenges of data scarcity, SynFit introduces a synergistic contrastive learning algorithm that effectively recalibrates pretrained pLM representations toward assay-specific fitness data while avoiding overfitting. Based on these predictions, SynFit prioritizes protein variants that simultaneously optimize multiple functional properties.

To evaluate SynFit, we curated a multi-fitness benchmark derived from ProteinGym^16^, comprising 46 deep mutational scanning (DMS) assays across 20 proteins and spanning diverse functions. Across this benchmark, SynFit consistently outperforms single-objective models trained on individual assays, identifies variants that balance multiple functional constraints, and reveals residues with shared functional importance across assays. We further validated SynFit experimentally in a new-to-nature biocatalytic borylation system catalyzed by a heme-dependent cytochrome c protein, demonstrating that SynFit-guided designs could furnish a diverse collection of novel combinatorial biocatalyst variants with catalytic activity and enantioselectivity that rival or surpass those obtained through laboratory directed evolution. Sequence analysis of top-performing variants revealed convergent residue preferences across the engineered active-site positions, consistent with a focused, multi-objective optimization of the combinatorial sequence space. Together, these results establish SynFit as a general framework for multi-objective protein fitness prediction and engineering and highlight its potential to advance ML–guided protein engineering, particularly for use in biocatalysis.

## 2 Results

### 2.1 Synergistic contrastive learning for multi-objective fitness prediction

**Figure 1:**
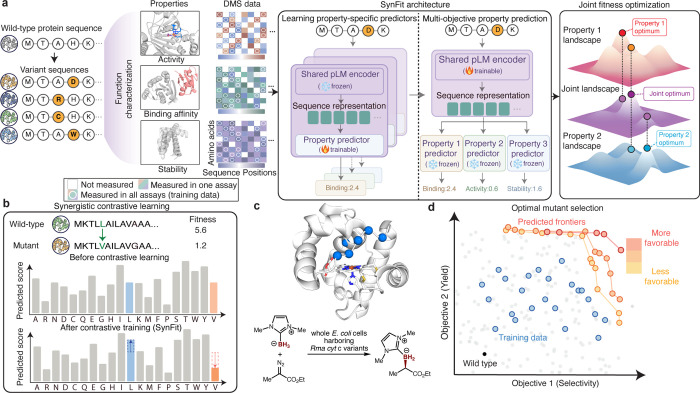
Overview of our multi-fitness prediction framework. **(a)** Protein variants derived from the wildtype sequence are experimentally characterized for a subset of properties to generate partial deep mutational scanning (DMS) data used for model training.SynFit jointly fine-tunes a pretrained protein language model across multiple assays using a shared encoder and property-specific predictors. **(b)** Each objective combines a contrastive BT loss with KL regularization against the ESM2 prior, and calibrated predictions are integrated via non-dominated sorting to yield multi-objective Pareto surfaces. **(c)** To validate SynFit, we applied it to the combinatorial optimization of *Rma* cyt c variants for asymmetric carbene B–H insertion, a new-to-nature biocatalytic reaction that converts NHC·BH_3_ and α-diazocarbonyl compounds to chiral organoboron products in whole *E. coli* cells. **(d)** SynFit-predicted multi-objective fitness landscape for 909 experimentally characterized *Rma* cyt c variants, with Pareto-optimal candidates selected for experimental validation.

Protein engineering often requires optimizing multiple functional properties simultaneously, such as activity, stability, or selectivity. These properties are frequently coupled: a mutation that improves one aspect of function may weaken another, whereas some mutations influence multiple properties through shared structural or biochemical mechanisms. In typical deep mutational scanning (DMS) experiments, however, each assay captures only one facet of protein properties, and different assays provide only partial and heterogeneous measurements of the same sequence landscape. To address this challenge, we developed SynFit, a framework that jointly learns from multiple functional assays to model the multidimensional fitness landscape of a protein (Fig. 1a).

SynFit starts from a pretrained pLM, which encodes broad evolutionary information from natural protein sequences. Building on this backbone architecture, SynFit learns a shared representation that captures sequence features relevant across functional readouts, together with property-specific predictors that model individual objectives (Fig. 1b). In this way, the framework captures both common constraints that shape multiple biochemical properties and the distinct signals associated with each assay, rather than treating each measurement independently. We experimentally validated SynFit on the combinatorial optimization of *Rma* cyt c variants for asymmetric carbene B–H insertion (Fig. 1c), a new-to-nature biocatalytic reaction optimized jointly for yield and enantioselectivity. The resulting predictions can then be used to prioritize variants that achieve the best overall balance across multiple objectives, which is essential for practical protein engineering applications (Fig. 1d).

This framework is designed to make multi-objective learning effective even when experimental data are limited. Rather than extensively retraining the pretrained model to fit absolute assay values, SynFit adapts it in a data-efficient manner by learning relative differences among variants within each assay. This strategy builds on our previous low-N fitness prediction model^23^ while advancing it to the multi-objective setting through joint learning across multiple functional assays. This strategy preserves the broad biological information encoded during pretraining while recalibrating the model toward experimentally measured function, thereby reducing overfitting to small and noisy datasets. Together, these design features enable SynFit to learn robust and biologically meaningful multidimensional fitness landscapes from sparse multi-assay measurements.

### 2.2 SynFit enables accurate and generalizable protein fitness prediction

To evaluate whether the multi-objective learning of SynFit improves protein fitness prediction, we curated a comprehensive benchmark of proteins with multiple DMS assays from ProteinGym^16^. Specifically, we combined individual single-property functional assays from various studies characterizing the same protein and retained overlapping single-mutation variants with quantitative measurements across all available assays, constructing a “multi-fitness” dataset for rigorous evaluation of multi-objective prediction. The resulting benchmark includes 46 assays across 20 proteins spanning diverse organisms, with each protein annotated by 2–4 functional properties (Table S1). This dataset enables a systematic assessment of multi-objective fitness prediction performance.

**Figure 2:**
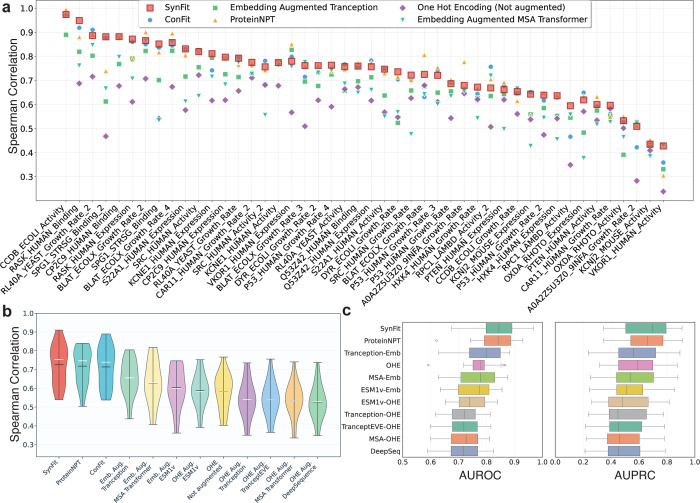
Comprehensive evaluation of SynFit function prediction performance across proteins and fitness assays. *Abbreviations:* OHE: one-hot encoding; emb.: embedding. **(a)** Five-fold cross-validation performance son held-out test data for top-performing methods, where SynFit demonstrates both high mean correlations and low variance, highlighting its robustness and generalizability across fitness dimensions. Each model was trained and evaluated over five random splits, and the reported values represent the average Spearman correlation between predicted and experimentally measured fitness across held-out test sets. **(b)** AUROC (left) and AUPRC (right) distributions on 13 proteins (28 DMS) with available wild-type anchors. SynFit achieves the highest overall performance, showing strong ability to identify beneficial mutants relative to wild type. **(c)** Distribution of protein-level Spearman correlations across the multi-fitness benchmark. SynFit achieves the highest average correlation across diverse proteins compared to all baselines.

We first tested whether synergistic learning across assays improves predictive accuracy compared to models trained on individual properties. SynFit was trained jointly on all available assays for each protein and evaluated on individual objectives, alongside established single-objective baselines. Across proteins, SynFit achieves the best performance on the majority of cases, demonstrating robustness across diverse sequence families (Fig. 2a). When aggregated across all assays, SynFit attains the highest mean and median Spearman correlations under five-fold cross-validation, outperforming strong baselines such as ConFit^23^ and ProteinNPT^32^ (Fig. 2b). These results indicate that multi-objective modeling across related functional measurements improves generalization and enables more accurate prediction of mutational effects across heterogeneous biochemical contexts.

Beyond correlation-based evaluation, a key objective in protein engineering is to identify variants that improve upon the wild type. To assess this capability, we evaluated models on 13 proteins labeled with explicit wild-type fitness values, which allows mutations to be binarized as beneficial or deleterious. SynFit consistently achieves the highest AUROC and AUPRC across proteins (Fig. 2c), indicating superior ability to prioritize beneficial variants from large mutational libraries.

Together, these results demonstrate that SynFit not only improves fitness prediction accuracy but also enhances the identification of functionally improved variants. By integrating complementary signals across multiple functional assays, the model more effectively captures sequence–function relationships that generalize across diverse sequence contexts.

### 2.3 SynFit identifies mutations that co-improve multiple properties

Models trained on individual properties often prioritize variants that perform well on one objective but poorly on others, limiting their ability to identify solutions that satisfy multiple functional constraints. To evaluate whether SynFit captures true cross-objective relationships, we assessed its ability to recover variants that simultaneously optimize multiple properties.

**Figure 3:**
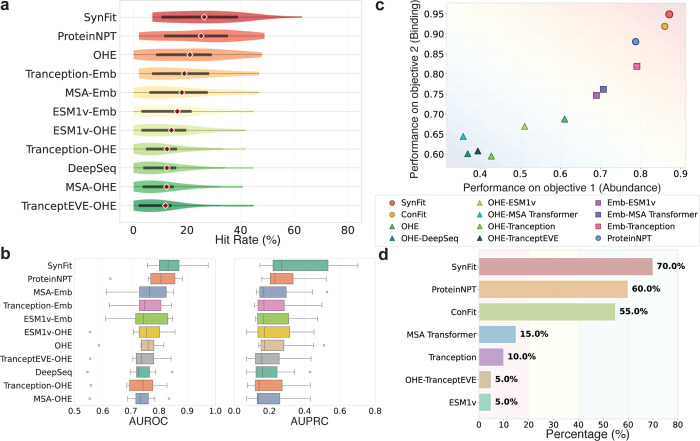
Multi-objective benchmarking of SynFit reveals synergistic optimization across fitness assays. *Abbreviations:* OHE: one-hot encoding. **(a)** Hit rate comparison of top 100 predicted versus ground-truth Pareto-optimal mutants, where each overlapping variant is counted as a “hit”, with SynFit achieving the highest overlap. **(b)** SynFit excels at identifying variants that improve upon wildtype across multiple fitness objectives, consistently enriching for jointly beneficial mutants. **(c)** Pareto analysis of held-out functional assays for *RASK_HUMAN*, based on averaged Spearman correlations across five test folds, where SynFit occupies the optimal front and outperforms all baselines. **(d)** Comparative Pareto analysis across proteins, using averaged Spearman correlations from five test folds; SynFit most frequently occupies the most optimal front.

We ranked variants based on their joint performance across objectives using non-dominated sorting, which organizes variants into Pareto fronts (Supplementary Note A.1). In this sorting process, variants are grouped such that those on the first Pareto front are not outperformed by any other variant when all properties are considered together. We then quantified performance using a hit-rate metric, defined as the overlap between the top 100 variants predicted by the model and the top 100 variants identified from experimental measurements. SynFit achieves the highest average hit rate across all proteins (Fig. 3a), indicating that joint learning enables more accurate identification of variants that co-optimize multiple properties than approaches based on independently trained models.

We next evaluated the ability of SynFit to identify jointly beneficial variants, defined as mutations that outperform the wild type across all measured objectives (Fig. 3b). This represents a stringent benchmark, as variants must improve all properties simultaneously. Across eight proteins spanning 16 DMS assays with available wild-type annotations (Table S1), SynFit consistently achieves higher AUROC and AUPRC than single-objective baselines, demonstrating improved ability to prioritize variants with multi-property gains.

To further illustrate SynFit’s multi-objective capability, we analyzed the *RASK_HUMAN* dataset (Fig. 3c), which includes abundance and binding assays. Models were evaluated based on their ability to achieve optimal trade-offs among these properties. SynFit is the only model that lies on the first Pareto front (Supplementary Note A.1), indicating that it successfully captures the joint effects of mutations on both abundance and binding. Extending this analysis across all proteins, SynFit appears on the first Pareto front for 70% of proteins, outperforming ProteinNPT (60%) and ConFit (55%) (Fig. 3d). These results further support that SynFit achieves both strong per-assay accuracy while capturing shared functional representations that provide Pareto-optimal predictions across diverse protein fitness landscapes.

### 2.4 In silico case study: SynFit identifies shared functional residues in KRAS across multiple binding partners

Proteins that interact with multiple binding partners often rely on a subset of residues that mediate binding across partially overlapping interfaces. Mutations at these sites can simultaneously alter multiple interactions, thereby reshaping protein interaction networks and functional activity. Identifying such shared interaction determinants is important for understanding mutational effects and for designing proteins with controlled interaction profiles. However, models trained on individual assays typically capture only binding partner-specific signals and cannot distinguish residues that broadly regulate multiple interactions. We thus tested whether SynFit’s multi-assay learning framework can more effectively identify residues that govern binding across multiple partners. This setting naturally calls for a multi-objective approach that integrates information across assays to uncover shared biophysical constraints beyond what can be learned from any single assay.

Mutational effects often arise through distinct mechanisms. Some mutations primarily affect folding or stability, whereas others directly perturb active or interaction sites. Disentangling these effects is challenging when each assay provides only a single view of protein function^34^. Our previous studies^26;34^ addressed this issue by quantifying mutation effects on function beyond what can be explained by stability alone, thereby identifying functional residues. Here, SynFit extends this principle to the multi-assay setting, where multiple functional measurements provide complementary information about shared and context-specific determinants. By jointly learning across assays, SynFit can identify residues whose effects are consistently functional across different interaction contexts.

**Figure 4:**
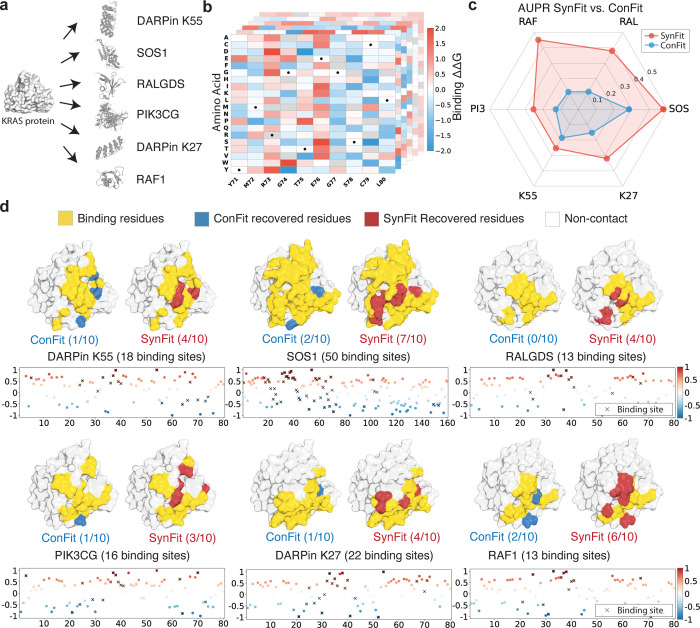
SynFit accurately identifies functional residues across KRAS binding partners by leveraging shared biophysical constraints. **(a)** We curated 6 DMS binding assays from KRAS binding DMS^33^ corresponding to 6 binding partners. **(b)** After preprocessing, we obtained 6 DMS datasets consisting of 2,418 variants each. (Black dots indicate the wild-type amino acid at each position) **(c)** SynFit achieves higher AUPR performance than individual ConFit model in identification of KRAS binding residues. **(d)** SynFit (bottom panel) performance indicates a substantial improvement compared to individual ConFit models (top panel) in both classification accuracy and positive predictive power for binding sites identification. Yellow highlights indicate partner-specific interface residues; red highlights mark residues contacting all six partners.

To assess whether SynFit can learn shared functional determinants across related interaction contexts, we applied it to KRAS, a central signaling protein that interacts with multiple downstream effector proteins (Fig. 4a). Recent deep mutational scanning (DMS) experiments profiled KRAS variants across six binding partners, with each assay quantitatively measuring how mutations affect binding affinity to a specific effector^33^. Because these assays probe overlapping interaction interfaces, a unified framework should better capture shared biophysical constraints than partner-specific models. We therefore trained SynFit jointly across all six DMS datasets and compared it with individual ConFit models trained separately on each binding partner (Fig. 4b). Final residue-level function scores were averaged across independent runs and evaluated by their ability to recover known binding and allosteric sites using AUPR (Methods). Interface sites were defined by a 5Å cutoff in PDB structures, and residues shared across all six partners were treated as common sites and used as the ground-truth positive samples.

We observed that the joint SynFit model consistently outperformed single-assay ConFit models in identifying functional residues, achieving higher AUPR across all binding partners (Figs. 4c,d). Multi-assay training exposed shared functional determinants, such as switch-region and nucleotide-pocket residues, enabling SynFit to disentangle stability-mediated effects from genuine functional disruptions. These results demonstrate that SynFit can uncover shared biophysical constraints governing multi-partner interactions, enabling systematic identification of residues with shared functional roles across multiple binding partners.

Beyond predictive performance, we examined the biological relevance of residues identified as shared across all six binding partners (Fig. 4d, red). These residues partially overlap with known regulatory regions of KRAS, including the switch I (residues 30–38) and switch II (residues 59–67) regions^35;36^, which mediate conformational changes and effector recognition.

Together, these results show that SynFit not only improves predictive accuracy but also reveals biologically important residues associated with shared functional mechanisms across interaction contexts, providing a valuable method for understanding mutational effects and guiding multi-objective protein design.

### 2.5 SynFit designs combinatorial enzymes with simultaneously optimized activity and enantioselectivity for new-to-nature biocatalysis

**Figure 5:**
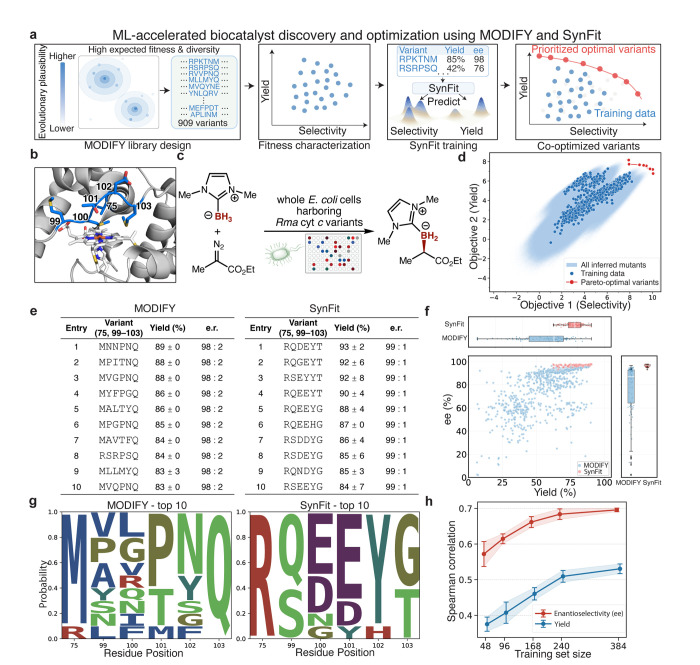
Experimental validation of SynFit for asymmetric carbene B–H insertion catalyzed by *Rma* cyt c variants. **(a)** Two-stage biocatalyst design workflow combining MODIFY library design with SynFit multi-objective optimization. **(b)** Structure of *Rhodothermus marinus* cytochrome c (PDB 6CUK) highlighting the six targeted active-site residues for combinatorial mutagenesis (positions 75, 99–103). **(c)** Asymmetric carbon–boron bond forming reaction catalyzed by *Rma* cyt c variants. Reaction conditions: *E. coli* cells overexpressing *Rma* cyt c in M9-N buffer (OD_600_ = 30, ca. 0.03–0.05 mol% *Rma* cyt c), NHC-BH_3_ (10 mM), ethyl diazoacetate (10 mM), 5% MeCN, 680 rpm, 12 h. **(d)** SynFit-predicted multi-objective landscape for the 909 experimentally characterized variants. Red points indicate Pareto-optimal *Rma* cyt c variants selected for experimental validation. **(e)** Comparison of the ten highest-yielding highly selective variants from the MODIFY and SynFit libraries. Top variants were defined within the highly selective regime and ranked by yield. **(f)** Experimental validation of the top 100 SynFit-predicted variants relative to the original MODIFY-derived dataset, highlighting enrichment of SynFit variants in the high-yield, high-selectivity region. **(g)** Sequence-logo comparison of top ten variants discovered from MODIFY (left) and SynFit (right) libraries exhibiting the highest activity and enantioselectivity, showing stronger sequence convergence among SynFit top variants across the six engineered positions. **(h)** Spearman correlation between predicted and experimentally measured enantioselectivity and yield as a function of training set size for *Rma* cyt c.

We developed a two-stage machine learning workflow that integrates computational library design with multi-objective fitness prediction for biocatalyst optimization (Fig. 5a). First, MODIFY^37^ designs a starting library balancing evolutionary plausibility with sequence diversity across six active-site positions. After experimental characterization of yield and enantioselectivity, SynFit is trained on these sequence–fitness pairs to predict both objectives across the combinatorial sequence space, and non-dominated sorting identifies Pareto-optimal designs for validation. To experimentally validate SynFit’s predictive capability for use in biocatalysis and enzyme engineering, we applied it to the combinatorial optimization of enzyme variants for biocatalytic asymmetric borylation using N-heterocyclic carbene-boranes (NHC·BH_3_) and α-diazocarbonyl compounds, a recently discovered enzymatic reaction ^38^ operating through a carbene transfer mechanism not present in the biological world (Figs. 5b,c). This borylation reaction converts readily accessible building blocks into chiral organoboron products with broad applications in medicinal chemistry and functional materials. We implemented SynFit to optimize biocatalysts derived from the wild-type *Rhodothermus marinus* cytochrome c (*Rma* cyt c), a compact heme protein consisting of 124 amino acid residues from a hyperthermophile. In optimizing enzyme variants for this reaction, two complementary objectives, overall catalytic yield and enantioselectivity, need to be simultaneously optimized, making it an excellent testing ground for evaluating whether SynFit can jointly model and optimize multiple functional properties. Guided by the crystal structure of *Rma* cyt c (PDB ID: 6CUK), we focused our efforts to generate highly active and enantioselective variants by optimizing amino acid residues 75 and 99–103 proximal to heme cofactor in the active site. We first trained SynFit on the 909 experimentally characterized hextuple variants generated by our previously developed method MODIFY^37^, an unsupervised ML–guided evolution-inspired starting-library design method that balances expected library fitness with sequence diversity. We constructed three non-overlapping train-validation splits and trained multiple SynFit models under different random seeds to ensemble their predictions (Methods). The aggregated predictions were then subjected to non-dominated sorting (Supplementary Note A.1) to identify Pareto-optimal variants (Fig. 5d). The top 100 SynFit-designed variants were produced as an oligo-pool encoding the diversified sequence region, assembled into full-length *Rma* cyt c expression constructs in pET-22b(+), and expressed in *Escherichia coli*. The activity and enantioselectivity of these SynFit-designed cytochrome c variants were assessed in whole-cell assays for the enantioselective C–B bond forming reaction.

Starting from the custom-synthesized oligo-pool library encoding SynFit-designed hextuple mutants, we carried out high-throughput biocatalytic borylation reaction using four 96-well plates. Each clone from the 96-well screening was verified by Sanger sequencing. For SynFit variants that appeared more than once in the screening library, catalytic activity and enantioselectivity data were consolidated and standard deviations were calculated to ensure the reliability of our high-throughput results. Together, this effort led to a new biocatalyst activity and enantioselectivity dataset covering a total of 83 variants out of the 100 designs. Across this experimentally validated dataset, SynFit-guided designs exhibited a clear shift toward simultaneously and substantially enhanced yields and enantioselectivities in the multidimensional fitness landscape (Fig. 5f), with a multitude of variants exhibiting superior catalytic performance than all MODIFY variants present in the training data. Unlike MODIFY, which was designed to construct evolution-inspired starting libraries enriched for fitness and sequence diversity, SynFit employs a supervised multi-objective optimization framework that quantitatively links sequence to both catalytic activity and enantioselectivity, enabling the discovery of *Rma* cyt c variants with superior catalytic performance beyond the starting library.

Beyond the overall shift in the library-level fitness landscape, we also examined the upper range of practically useful biocatalyst performance among the experimentally validated SynFit variants. Because a range of top-performing *Rma* cyt c SynFit variants displayed excellent enantioselectivities up to 99:1 e.r., we ranked these highly enantioselective SynFit variants primarily by yield. It was found that the top 10 SynFit variants all reached yield greater than 84% with an enantioselectivity of 99:1 e.r. (Fig. 5e). Thus, SynFit discovered a range of combinatorial borylation biocatalysts with catalytic performance similar to the experimentally evolved *Rma* cyt c resulting from three rounds of site-saturation mutagenesis and screening^38^.

We next asked whether these top-performing SynFit variants also shared common sequence features. Sequence analysis of the experimentally validated top variants revealed that the principal difference between the two libraries was not only in performance, but also in sequence convergence across the six engineered active-site positions. Notably, SynFit recapitulates key beneficial features of the experimentally evolved variant^38^, including R75 and T103, while prioritizing a carboxylate side chain (glutamate (E) and aspartate (D)) at residue 100. Wild-type *Rma* cyt c features a methionine (M) at residue 100 as a chelator of the heme cofactor. Thus, the preference for E and D at residue 100 reveals a distinctive feature enriched in top-performing borylation biocatalysts, which is also in accord with the experimentally evolved variant^38^. More interestingly, SynFit also uncovers additional convergent preferences, including Q99, E101, and Y102 at these sites, which diverge from the experimentally evolved variant. In contrast, the top variants from MODIFY were substantially more heterogeneous at these residues (Fig. 5g). This result suggests that SynFit does not merely retrieve isolated high-performing variants, but instead focuses the combinatorial landscape toward a narrower set of residue combinations associated with jointly improved catalytic activity and stereoselectivity. Together, these findings show that SynFit transforms generative, evolution-inspired starting libraries into multi-objective-optimized design spaces, enabling the discovery of new-to-nature biocatalysts with improved activity and selectivity.

In real-world enzyme engineering efforts, due to the limited throughput in chromography-based activity and stereoselectivity screening, experimental datasets are often of the size of 10^2^ variants, making it critical for predictive machine learning models to perform effectively with small training sets. To evaluate the utility of SynFit in biocatalysis research, we examined its performance in data-limited regimes. We performed a low-N scaling analysis by subsampling the training set of *Rma* cyt c variants to sizes ranging from 48 to 384 variants (Fig. 5h). SynFit was trained independently on each subset and evaluated on held-out variants using Spearman correlation for both yield and enantioselectivity. Notably, SynFit retained strong predictive accuracy even when trained on fewer than 100 labeled variants, while performance improved steadily as additional data became available. These results highlight the practical utility of SynFit for guiding enzyme engineering in laboratory settings with modestly sized enzyme activity and stereoselectivity datasets.

## 3 Discussion

Protein function is inherently multi-dimensional. A mutation can simultaneously affect stability, activity, binding, expression, or selectivity, and these effects are often mechanistically coupled rather than independent. This coupling is particularly important in protein engineering, where desirable variants rarely optimize only one property in isolation. In many application domains, particularly in biocatalysis and enzyme engineering, the need to simultaneously optimize multiple functional properties, most prominently catalytic activity and selectivity, represents a pervasive and central challenge. These objectives are often interdependent, making the identification of optimal variants difficult using approaches that treat each property independently.

In this work, we present SynFit, a multi-objective protein fitness prediction framework designed to model this interconnected nature of protein function. Our results show that explicitly capturing relationships among functional properties improves the prediction of multidimensional mutation effects and the identification of variants that co-optimize multiple objectives. A central insight of SynFit is that multiple functional assays measured on the same protein contain complementary views of a shared underlying sequence-function landscape. Mutations that disrupt folding can broadly affect abundance, binding, or catalytic activity, whereas mutations at functional sites may selectively impact specific properties. By jointly learning across related objectives, SynFit captures both common constraints critical across multiple functions and objective-specific effects that shape individual functional readouts. The consistent improvements over independently trained models suggest that these cross-assay relationships are biologically meaningful and can be effectively exploited for protein fitness prediction.

This perspective further underscores the importance of multi-objective fitness modeling in guiding protein engineering. In practical settings, the goal is rarely to optimize a single metric, but rather to identify variants that balance multiple functional requirements. This is particularly important in biocatalysis, where achieving high catalytic turnover while furnishing excellent chemo-, regio- and stereoselectivity remains a fundamental challenge. Our benchmark results show that SynFit more effectively prioritizes such variants, indicating that multi-objective learning provides a more holistic representation of protein fitness landscapes than single-property approaches.

The experimental validation in the *Rma* cyt c borylation system provides an excellent test case for this capability. In this new-to-nature enzymatic reaction, catalytic activity and enantioselectivity must be simultaneously optimized, reflecting a common constraint in biocatalyst development. SynFit enabled the discovery of a diverse set of cytochrome c variants, including numerous sextuple mutants, which exhibit substantial and coordinated improvements in both catalytic activity and stereoselectivity. Notably, these variants populate the high-performance region of the multidimensional fitness landscape and achieve levels of activity and selectivity rivaling or superior than those obtained through multiple rounds of experimental directed evolution. These results highlight SynFit as an effective strategy for navigating complex combinatorial sequence spaces and for identifying practically useful biocatalysts under realistic multi-objective constraints.

Another key aspect of SynFit is its integration of evolutionary priors from pretrained protein language models (pLMs) with assay-specific experimental measurements. Pretrained pLMs capture broad patterns of evolutionary sequence plausibility but do not directly encode functional objectives. SynFit adapts these general representations into function-specific predictive models using experimental fitness data, while preserving their generalizable feature. This enables robust performance even when labeled data are limited, a common scenario in enzyme engineering where throughput constraints restrict dataset size. Together, these design choices make SynFit particularly well suited for real-world multi-objective protein engineering tasks.

Beyond predictive performance, SynFit also offers insights into the molecular mechanisms underlying protein function. When trained across related assays, the model can highlight residues whose mutation effects are consistent across functional measurements, revealing their shared structural or mechanistic constraints. This was illustrated in the KRAS case study, where joint modeling across binding assays enhanced the identification of residues with functional importance across multiple binding partners. Such analyses suggest that multi-objective learning can help distinguish broadly important functional sites from assay-specific effects, extending SynFit’s utility from variant effect prediction to mechanistic interpretation.

Several opportunities remain for further development. Many experimental datasets contain only partial overlap across measured properties, and extending SynFit to more flexibly handle partially overlapped data would broaden its applicability. In addition, while the current SynFit framework relies primarily on sequence-based representations, many functional trade-offs are mediated by structural context and conformational dynamics. Incorporating richer structural information may further improve the modeling of coupled biochemical properties.

Overall, our results support a broader view of protein fitness prediction: multi-assay measurements that characterize different functions of the same protein can be combined to learn more informative fitness prediction models. By enabling the identification of variants that simultaneously improve multiple properties, SynFit provides a general framework for addressing a longstanding challenge in protein engineering. As high-throughput mutational data continue to expand across assays and properties, we envision SynFit as a useful tool for mechanistic studies of protein function and ML-guided protein engineering.

## 4 Methods

### 4.1 Protein Language Models for Protein Fitness Prediction

Recent advances in large-scale protein language models (pLMs)^39–42^ have demonstrated that amino acid sequences can be modeled analogously to natural language. These models are trained in an unsupervised fashion on massive databases of natural sequences such as UniProt^43^. Most state-of-the-art pLMs, including ESM3^41^, ProGen3^44^, and ProGen2^13^ adopt a masked language modeling (MLM) objective in which the model is trained to predict masked residues $x_{i}$ given the surrounding context $x_{-i}$. Formally, the training objective maximizes the conditional log-likelihood ${𝓛}_{\text{MLM}}=\sum_{i\in 𝒮}\log p_{\theta }\left(x_{i}|x_{-i}\right)$, where 𝒮 denotes the set of masked positions. Through pretraining, pLMs capture patterns of residue co-occurrence that reflect evolutionary constraints on protein sequences, providing a notion of how plausible a given mutation is within the space of natural proteins. As a result, zero-shot mutational scoring from pLMs has been shown to correlate with DMS measurements by quantifying the log-likelihood difference between wild-type and mutant residues. However, such unsupervised training does not incorporate function-specific information, which often depends on subtle biochemical determinants beyond evolutionary plausibility. This motivates supervised adaptation strategies, such as ConFit^23^ and ProteinNPT^32^, to steer pretrained pLMs toward protein-specific functional objectives. Because these models are trained on large-scale evolutionary data, previous research^45;46^ has demonstrated the log-likelihood ratio between a wild-type residue and its mutant counterpart provides a useful proxy for relative fitness. Formally, for a mutation $x_{i}^{WT}\to x_{i}^{MT}$, the pLM-derived score is

$$s_{\theta_{0}}\left(x^{MT};i\right)=\log p_{\theta_{0}}\left(x_{i}^{MT}|x_{-i}^{MT}\right)-\log p_{\theta_{0}}\left(x_{i}^{WT}|x_{-i}^{WT}\right).$$

Aggregating across sites yields a zero-shot estimate of mutant plausibility.

Traditionally, supervised fine-tuning approaches such as ConFit^23^ and ProteinNPT^32^ focus on optimizing a single functional objective y∈ℝ (e.g., binding affinity or catalytic activity). Formally, given training data ${\left\{\left(x^{\left(k\right)},y_{k}\right)\right\}}_{k=1}^{N}$, the goal is to learn a mapping $f:{𝒳}^{L}\to \mathbb{R}$, $\hat{y}(x)\approx y$, where x is a protein sequence and y is its corresponding functional measurement. However, in many practical protein engineering settings, multiple properties are simultaneously relevant. For instance, a mutation that improves catalytic yield may reduce thermostability, while a destabilized protein often fails to fold correctly and thus cannot carry out its biological function. Therefore, we are interested in learning predictive models that can jointly optimize multiple objectives and capture their trade-offs. We denote the set of M quantitative objectives as $𝒴=\left\{y^{\left(1\right)},y^{\left(2\right)},\ldots ,y^{\left(M\right)}\right\}$, $y^{\left(m\right)}\in \mathbb{R}$. Given supervised training data now consists of ${\left\{\left(x^{\left(k\right)},y_{k}^{\left(1\right)},\ldots ,y_{k}^{\left(M\right)}\right)\right\}}_{k=1}^{N}$, where each $x^{\left(k\right)}$ is a variant of the wild-type sequence and $y_{k}^{\left(m\right)}$ is its experimentally measured fitness under objective m. Our aim is to learn a multi-objective model $f:{𝒳}^{L}\to {\mathbb{R}}^{M}$, $\hat{y}(x)=\left({\hat{y}}^{\left(1\right)}(x),\ldots ,{\hat{y}}^{\left(M\right)}(x)\right)$, such that each ${\hat{y}}^{\left(m\right)}(x)$ provides an accurate prediction of the true experimental measurement $y^{\left(m\right)}$. By sharing a common representation across tasks while retaining task-specific prediction heads, SynFit leverages correlations between objectives to improve predictive accuracy and robustness.

### 4.2 Overall Training Pipeline

#### Multi-fitness benchmark.

We curated a multi-fitness benchmark from ProteinGym^16^, retaining proteins with multiple functional assays to enable cross-objective evaluation. Datasets with insufficient variant coverage or sequences exceeding GPU memory limits were excluded. The final benchmark comprises 46 DMS assays across 20 proteins, spanning diverse molecular functions including binding, stability, catalysis, and expression.

#### Stage-wise training.

To balance task specialization and cross-objective generalization, SynFit employs a novel two-stage training procedure: *Independent head training:* Each task-specific head Head*_m_* is first trained separately on its corresponding deep mutational scanning (DMS) dataset, while keeping both the pretrained encoder $f_{\theta }$ and the shared module $g_{\phi }$ frozen. This ensures that each head captures the fitness signal specific to its objective without interference from other tasks. *Joint fine-tuning:* Once initialized, the heads are frozen and the shared module $g_{\phi }$ is trained jointly across all tasks. Gradients from all objectives flow into $g_{\phi }$, encouraging it to extract representations that are informative across correlated objectives.

#### Contrastive learning with BT loss and KL regularization.

For each objective m, we adopt the BT model^47^, which encourages correct ranking of variants. Given all pairs $\left(x_{i},x_{j}\right)$ such that $y_{i}^{\left(m\right)}>y_{j}^{\left(m\right)}$, the contrastive loss is

$${𝓛}_{BT}^{\left(m\right)}=\sum_{y_{i}^{\left(m\right)}>y_{j}^{\left(m\right)}}\log\left(1+\exp\left(-\left[{\hat{y}}^{\left(m\right)}\left(x_{i}\right)-{\hat{y}}^{\left(m\right)}\left(x_{j}\right)\right]\right)\right).$$

This formulation converts N labeled variants into $𝒪\left(N^{2}\right)$ comparisons, making efficient use of limited DMS data. To prevent the fine-tuned model from diverging too far from the pretrained distribution, we introduce a KL penalty between the predicted amino acid distribution of each head and that of the frozen pLM on the wild-type sequence $x^{WT}$. Let $p_{\text{pre }}\left(\cdot |x^{WT}\right)$ be the residue distribution from the pretrained model and $p_{\theta_{m}}\left(\cdot |x^{WT}\right)$ the distribution induced by head m. The KL regularizer is

$${𝓛}_{KL}^{\left(m\right)}=D_{KL}\left(p_{\text{pre }}\left(\cdot |x^{WT}\right)\|p_{\theta_{m}}\left(\cdot |x^{WT}\right)\right).$$

This regularization stabilizes training by anchoring predictions to the evolutionary prior encoded in the pretrained model, reducing overfitting to small-scale experimental datasets.

#### Per-task and overall objective.

The training objective for each task combines contrastive supervision and KL regularization:

$${𝓛}^{\left(m\right)}={𝓛}_{BT}^{\left(m\right)}+\lambda_{KL}{𝓛}_{KL}^{\left(m\right)},$$

where $\lambda_{KL}$ controls the strength of regularization. The global multi-task loss aggregates across objectives:

$$𝓛=\sum_{m=1}^{M}\alpha_{m}{𝓛}^{\left(m\right)},\;\alpha_{m}\geq 0,\;\sum_{m=1}^{M}\alpha_{m}=1.$$

In practice, $\alpha_{m}$ can be set to equal weights $\left(\alpha_{m}=1/M\right)$ or tuned to emphasize specific objectives.

### 4.3 Function Site Identification

We adapt the methodology of our previous SPURS model^26^ to identify functionally important sites, replacing ESM1v sequence-likelihood scores with the task-specific prediction heads of SynFit, while retaining SPURS-predicted ΔΔG as the stability reference signal. For a wild-type protein sequence ${\text{x}}^{\text{WT}}$ and a single-residue mutation $x_{i}^{\text{MT}}$ at position i, SynFit produces task-specific prediction scores

(1) $$f^{\left(k\right)}\left(i,x_{i}^{\text{MT}}\right)={\hat{y}}^{\left(k\right)}\left(x_{i}^{\text{MT}}|{\text{x}}^{\text{WT}}\right),\;k\in \{1,\ldots ,K\},$$

where K=6 for the six KRAS binding partners and $x_{i}^{\text{MT}}$ is the substituted amino acid. These scores serve as our proxy for the relative functional fitness of each single-mutation variant under binding partner k. We use SPURS^26^ to predict the folding free-energy change ΔΔG for every single-residue substitution and define a per-variant stability score as the clip-and-normalised negative SPURS-predicted ΔΔG,

(2) $$s_{\text{stab}}\left(i,x_{i}^{\text{MT}}\right)=-\varphi \left(\text{ΔΔ}G\left(x_{i}=x_{i}^{\text{MT}}|{\text{x}}^{\text{WT}}\right)\right),$$

where φ(⋅) clips values to the 0.1^th^ and 99.9^th^ percentiles of the ΔΔG distribution and rescales to [0,1], so that stabilizing mutations receive high scores and destabilizing ones receive low scores. To disentangle stability effects from binding-specific contributions, we fit a sigmoid function mapping SynFit prediction scores to SPURS stability scores across all single-mutation variants of each protein:

(3) $${\hat{s}}_{\text{stab}}\left(i,x_{i}^{\text{MT}}\right)=\sigma \left(\frac{f^{\left(k\right)}\left(i,x_{i}^{\text{MT}}\right)-\mu }{\tau }\right),$$

where μ and τ are location and scale parameters estimated by weighted least squares and σ(⋅) denotes the sigmoid function. The deviation of the observed stability score from the sigmoid prediction,

(4) $$\epsilon^{\left(k\right)}\left(i,x_{i}^{\text{MT}}\right)=s_{\text{stab}}\left(i,x_{i}^{\text{MT}}\right)-{\hat{s}}_{\text{stab}}\left(i,x_{i}^{\text{MT}}\right),$$

indicates whether a mutation’s stability effect is larger or smaller than expected from its functional score^48^. We summarise per-site importance as the *function score*: the mean residual across all substitutions at residue i,

(5) $$s_{\text{func}}^{\left(k\right)}(i)=\frac{1}{\left|{𝓜}_{i}\right|}\sum_{x_{i}^{\text{MT}}\in {𝓜}_{i}}\epsilon^{\left(k\right)}\left(i,x_{i}^{\text{MT}}\right),$$

where ${𝓜}_{i}$ is the set of all 19 non-wildtype amino acid substitutions at position i.

To curate our training dataset, we removed variants containing stop codons or higher order mutations, and retained only variants with complete measurements across all 6 binding assays, resulting in a total of 2,418 variants. During training, we employed a three–fold data-splitting strategy to ensure robust model evaluation and complete coverage of the dataset. For each fold, 90% of the data were randomly assigned to the training set and the remaining 10% were held out as the validation set. Three independent models were trained on these non-overlapping splits, such that each data point appeared in the validation set exactly once across the three folds. This design ensures that the combined set of validation predictions covers the entire dataset without redundancy.

While individually trained ConFit models achieve strong performance on their respective DMS assays, SynFit further improves upon these single-task models. By jointly training SynFit on all 6 binding DMS datasets across all binding partners (DARPin K55 (PDB ID: 5O2T, SOS1 (PDB ID: 1NVW), RALGDS (PDB ID: 1LFD), PIK3CG (PDB ID: 1HE8), DARPin K27 (PDB ID: 5O2S), and RAF1 (PDB ID: 6VJJ).), SynFit leverages mutual information shared across assays - for example, the critical common contact residues that contribute to multiple binding interfaces. This joint training enables SynFit to refine the function scores $s_{\text{func }}^{\left(k\right)}(i)$ and more accurately identify residues consistently under multi-partner functional constraint. The fact that SynFit outperforms all six ConFit baselines demonstrates that it effectively integrates information across shared binding sets, capturing functional signals that are not accessible to individually optimized models.

### 4.4 Application of SynFit to engineering *Rma* cyt c biocatalysts for carbene C–B bond formation

SynFit is experimentally validated on biocatalytic C–B bond-formation reaction catalyzed by *Rhodothermus marinus* cytochrome c (*Rma* cyt c). Building on the previously reported MODIFY-designed^37^ *Rma* cyt c library, we expanded experimental coverage from the initially tested 160 variants to a total of 909 sequence–function pairs. The designed sequences were synthesized as an oligo-pool library, cloned into a pET-22b(+) vector, and co-expressed with the heme-maturation plasmid pEC86 in *E. coli* BL21(DE3). Single colonies were picked into 96-well plates for small-scale expression under identical IPTG and 5-aminolevulinic acid induction at 20 °C. Following expression, cells were collected by centrifugation, resuspended in M9–N buffer, and directly used as whole-cell catalysts for the carbene C–B bond-formation reaction in 96-well plates under anaerobic conditions using N-heterocyclic carbene borane and ethyl diazoacetate as substrates. After 12 h, reactions were quenched and analyzed by chiral HPLC to determine total yield and enantioselectivity (e.r.). In parallel, the variants were characterized by PacBio long-read sequencing to verify sequence identity and ensure accurate sequence–function correspondence across the dataset. This standardized workflow produced a consistent, comparable dataset comprising 468 sequences from the intended MODIFY library and 441 additional recombined or non-designed variants across the same six active-site positions, providing a comprehensive experimental foundation for SynFit training and evaluation.

During training, we employed a three-split validation strategy to ensure both robustness and full dataset coverage. The 909 MODIFY variants were partitioned into three non-overlapping validation subsets, each comprising 10% of the data, while the remaining 90% served as the training set for that split. This ensured that every variant appeared in the validation set exactly once across the three runs. To further enhance robustness and account for stochastic variation, the full three-split procedure was repeated under five independent random seeds, resulting in a total of fifteen trained SynFit models. During inference, we averaged the predicted scores from all models to obtain the ensemble output for both enantioselectivity and yield. These ensemble predictions were ranked using non-dominated (Pareto) sorting, with the mean of both fitness scores resolving ties. (Supp. MethodsA.1) Because exhaustively sorting all possible mutants is not computationally realistic, we first identified a high-confidence candidate set by selecting variants that exceeded the 99.5th percentile of predicted scores in both objectives. All variants meeting this dual-percentile criterion were retained for non-dominated sorting. In this way, the 100 highest-ranking variants were selected for oligo-pool synthesis and experimental characterization.

## Supplementary Material

Supplement 1

## Data Availability

All code and data necessary to reproduce the results in this study are publicly available at: https://github.com/luo-group/SynFit. Additional materials are available from the corresponding author upon reasonable request.

## References

[1] Schymkowitz Joost, Borg Jesper, Stricher Francois, Nys Robby, Rousseau Frederic, and Serrano Luis. The foldx web server: an online force field. Nucleic acids research, 33(suppl 2):W382–W388, 2005. 15980494 10.1093/nar/gki387 PMC1160148

[2] Alford Rebecca F, Leaver-Fay Andrew, Jeliazkov Jeliazko R, O’Meara Matthew J, DiMaio Frank P, Park Hahnbeom, Shapovalov Maxim V, Renfrew P Douglas, Mulligan Vikram K, Kappel Kalli, The rosetta all-atom energy function for macromolecular modeling and design. Journal of chemical theory and computation, 13(6):3031–3048, 2017. 28430426 10.1021/acs.jctc.7b00125 PMC5717763

[3] Yang Kevin K, Wu Zachary, and Arnold Frances H. Machine-learning-guided directed evolution for protein engineering. Nature methods, 16(8):687–694, 2019. 31308553 10.1038/s41592-019-0496-6

[4] Luo Yunan, Jiang Guangde, Yu Tianhao, Liu Yang, Vo Lam, Ding Hantian, Su Yufeng, Qian Wesley Wei, Zhao Huimin, and Peng Jian. Ecnet is an evolutionary context-integrated deep learning framework for protein engineering. Nature communications, 12(1):5743, 2021. 10.1038/s41467-021-25976-8 PMC8484459 34593817

[5] Wittmann Bruce J, Johnston Kadina E, Wu Zachary, and Arnold Frances H. Advances in machine learning for directed evolution. Current opinion in structural biology, 69:11–18, 2021. 33647531 10.1016/j.sbi.2021.01.008

[6] Freschlin Chase R, Fahlberg Sarah A, and Romero Philip A. Machine learning to navigate fitness landscapes for protein engineering. Current opinion in biotechnology, 75:102713, 2022. 10.1016/j.copbio.2022.102713 PMC9177649 35413604

[7] Kouba Petr, Kohout Pavel, Haddadi Faraneh, Bushuiev Anton, Samusevich Raman, Sedlar Jiri, Damborsky Jiri, Pluskal Tomas, Sivic Josef, and Mazurenko Stanislav. Machine learning-guided protein engineering. ACS catalysis, 13(21):13863–13895, 2023. 37942269 10.1021/acscatal.3c02743 PMC10629210

[8] Meier Joshua, Rao Roshan, Verkuil Robert, Liu Jason, Sercu Tom, and Rives Alex. Language models enable zero-shot prediction of the effects of mutations on protein function. Advances in neural information processing systems, 34:29287–29303, 2021.

[9] Rao Roshan M, Liu Jason, Verkuil Robert, Meier Joshua, Canny John, Abbeel Pieter, Sercu Tom, and Rives Alexander. Msa transformer. In International conference on machine learning, pages 8844–8856. PMLR, 2021.

[10] Hie Brian, Zhong Ellen D, Berger Bonnie, and Bryson Bryan. Learning the language of viral evolution and escape. Science, 371(6526):284–288, 2021. 33446556 10.1126/science.abd7331

[11] Hie Brian L, Yang Kevin K, and Kim Peter S. Evolutionary velocity with protein language models predicts evolutionary dynamics of diverse proteins. Cell Systems, 13(4):274–285, 2022. 35120643 10.1016/j.cels.2022.01.003

[12] Lin Zeming, Akin Halil, Rao Roshan, Hie Brian, Zhu Zhongkai, Lu Wenting, Smetanin Nikita, Verkuil Robert, Kabeli Ori, Shmueli Yaniv, Evolutionary-scale prediction of atomic-level protein structure with a language model. Science, 379(6637):1123–1130, 2023. 36927031 10.1126/science.ade2574

[13] Nijkamp Erik, Ruffolo Jeffrey A, Weinstein Eli N, Naik Nikhil, and Madani Ali. Progen2: exploring the boundaries of protein language models. Cell systems, 14(11):968–978, 2023. 37909046 10.1016/j.cels.2023.10.002

[14] Sun Ning, Zou Shuxian, Tao Tianhua, Mahbub Sazan, Li Dian, Zhuang Yonghao, Wang Hongyi, Cheng Xingyi, Song Le, and Xing Eric P. Mixture of experts enable efficient and effective protein understanding and design. bioRxiv, pages 2024–11, 2024.

[15] Li Mingchen, Tan Yang, Ma Xinzhu, Zhong Bozitao, Yu Huiqun, Zhou Ziyi, Ouyang Wanli, Zhou Bingxin, Tan Pan, and Hong Liang. Prosst: Protein language modeling with quantized structure and disentangled attention. Advances in Neural Information Processing Systems, 37:35700–35726, 2024.

[16] Notin Pascal, Kollasch Aaron, Ritter Daniel, Van Niekerk Lood, Paul Steffanie, Spinner Han, Rollins Nathan, Shaw Ada, Orenbuch Rose, Weitzman Ruben, Proteingym: Large-scale benchmarks for protein fitness prediction and design. Advances in Neural Information Processing Systems, 36:64331–64379, 2023.

[17] Schmirler Robert, Heinzinger Michael, and Rost Burkhard. Fine-tuning protein language models boosts predictions across diverse tasks. Nature Communications, 15(1):7407, 2024. 10.1038/s41467-024-51844-2 PMC11358375 39198457

[18] Chu Simon KS, Narang Kush, and Siegel Justin B. Protein stability prediction by fine-tuning a protein language model on a mega-scale dataset. PLoS computational biology, 20(7):e1012248, 2024. 10.1371/journal.pcbi.1012248 PMC11293664 39038042

[19] Sledzieski Samuel, Kshirsagar Meghana, Baek Minkyung, Dodhia Rahul, Ferres Juan Lavista, and Berger Bonnie. Democratizing protein language models with parameter-efficient fine-tuning. Proceedings of the National Academy of Sciences, 121(26):e2405840121, 2024. 10.1073/pnas.2405840121 PMC11214071 38900798

[20] Zhou Ziyi, Zhang Liang, Yu Yuanxi, Wu Banghao, Li Mingchen, Hong Liang, and Tan Pan. Enhancing efficiency of protein language models with minimal wet-lab data through few-shot learning. Nature Communications, 15(1):5566, 2024. 10.1038/s41467-024-49798-6 PMC11219809 38956442

[21] Blalock Nathaniel, Seshadri Srinath, Babbar Agrim, Fahlberg Sarah A, Kulkarni Ameya, and Romero Philip A. Functional alignment of protein language models via reinforcement learning. bioRxiv, pages 2025–05, 2025.

[22] Biswas Surojit, Khimulya Grigory, Alley Ethan C, Esvelt Kevin M, and Church George M. Low-n protein engineering with data-efficient deep learning. Nature methods, 18(4):389–396, 2021. 33828272 10.1038/s41592-021-01100-y

[23] Zhao Junming, Zhang Chao, and Luo Yunan. Contrastive fitness learning: Reprogramming protein language models for low-n learning of protein fitness landscape. In International Conference on Research in Computational Molecular Biology, pages 470–474. Springer, 2024.

[24] Widatalla Talal, Rafailov Rafael, and Hie Brian. Aligning protein generative models with experimental fitness via direct preference optimization. bioRxiv, pages 2024–05, 2024.

[25] Tan Yang, Li Mingchen, Zhou Bingxin, Zhong Bozitao, Zheng Lirong, Tan Pan, Zhou Ziyi, Yu Huiqun, Fan Guisheng, and Hong Liang. Simple, efficient, and scalable structure-aware adapter boosts protein language models. Journal of Chemical Information and Modeling, 64(16):6338–6349, 2024. 39110130 10.1021/acs.jcim.4c00689

[26] Li Ziang and Luo Yunan. Generalizable and scalable protein stability prediction with rewired protein generative models. Nature Communications, 2025. 10.1038/s41467-025-67609-4 PMC12830971 41422228

[27] Luo Jiaqi, Ding Kerr, and Luo Yunan. Pareto-optimal sampling for multi-objective protein sequence design. iScience, 28(3), 2025. 10.1016/j.isci.2025.112119 PMC11952807 40160427

[28] Judy MV, Ravichandran KS, and Murugesan K. A multi-objective evolutionary algorithm for protein structure prediction with immune operators. Computer methods in biomechanics and biomedical engineering, 12(4):407–413, 2009. 19172428 10.1080/10255840802649715

[29] Suárez María, Tortosa Pablo, Carrera Javier, and Jaramillo Alfonso. Pareto optimization in computational protein design with multiple objectives. Journal of computational chemistry, 29(16):2704–2711, 2008. 18496793 10.1002/jcc.20981

[30] Bedbrook Claire N, Yang Kevin K, Robinson J Elliott, Mackey Elisha D, Gradinaru Viviana, and Arnold Frances H. Machine learning-guided channelrhodopsin engineering enables minimally invasive optogenetics. Nature methods, 16(11):1176–1184, 2019. 31611694 10.1038/s41592-019-0583-8 PMC6858556

[31] Silverstein Rachel A, Kim Nahye, Kroell Ann-Sophie, Walton Russell T, Delano Justin, Butcher Rossano M, Pacesa Martin, Smith Blaire K, Christie Kathleen A, Ha Leillani L, Custom crispr—cas9 pam variants via scalable engineering and machine learning. Nature, pages 1–3, 2025. 10.1038/s41586-025-09021-y PMC12449813 40262634

[32] Notin Pascal, Weitzman Ruben, Marks Debora, and Gal Yarin. Proteinnpt: Improving protein property prediction and design with non-parametric transformers. Advances in Neural Information Processing Systems, 36:33529–33563, 2023.

[33] Weng Chenchun, Faure Andre J, Escobedo Albert, and Lehner Ben. The energetic and allosteric landscape for kras inhibition. Nature, 626(7999):643–652, 2024. 38109937 10.1038/s41586-023-06954-0 PMC10866706

[34] Ding Kerr, Li Ziang, Tu Tony, Luo Jiaqi, and Luo Yunan. Deconvolving mutation effects on protein stability and function with disentangled protein language models. bioRxiv, 2026. doi: 10.64898/2026.02.03.703560. URL https://www.biorxiv.org/content/early/2026/02/26/2026.02.03.703560.

[35] Kim Hyeon Jin, Lee Han Na, Jeong Mi Suk, and Jang Se Bok. Oncogenic kras: signaling and drug resistance. Cancers, 13(22):5599, 2021. 34830757 10.3390/cancers13225599 PMC8616169

[36] Kirchhausen Tomas. Three ways to make a vesicle. Nature reviews Molecular cell biology, 1(3):187–198, 2000. 11252894 10.1038/35043117

[37] Ding Kerr, Chin Michael, Zhao Yunlong, Huang Wei, Mai Binh Khanh, Wang Huanan, Liu Peng, Yang Yang, and Luo Yunan. Machine learning-guided co-optimization of fitness and diversity facilitates combinatorial library design in enzyme engineering. Nature Communications, 15(1):6392, 2024. 10.1038/s41467-024-50698-y PMC11289365 39080249

[38] Kan SB Jennifer, Huang Xiongyi, Gumulya Yosephine, Chen Kai, and Arnold Frances H. Genetically programmed chiral organoborane synthesis. Nature, 552(7683):132–136, 2017. 29186119 10.1038/nature24996 PMC5819735

[39] Madani Ali, Krause Ben, Greene Eric R, Subramanian Subu, Mohr Benjamin P, Holton James M, Olmos Jose Luis Jr, Xiong Caiming, Sun Zachary Z, Socher Richard, Large language models generate functional protein sequences across diverse families. Nature biotechnology, 41(8):1099–1106, 2023. 10.1038/s41587-022-01618-2 PMC10400306 36702895

[40] Brandes Nadav, Goldman Grant, Wang Charlotte H, Ye Chun Jimmie, and Ntranos Vasilis. Genome-wide prediction of disease variant effects with a deep protein language model. Nature Genetics, 55(9): 1512–1522, 2023. 37563329 10.1038/s41588-023-01465-0 PMC10484790

[41] Hayes Thomas, Rao Roshan, Akin Halil, Sofroniew Nicholas J, Oktay Deniz, Lin Zeming, Verkuil Robert, Tran Vincent Q, Deaton Jonathan, Wiggert Marius, Simulating 500 million years of evolution with a language model. Science, 387(6736):850–858, 2025. 39818825 10.1126/science.ads0018

[42] Su Jin, Han Chenchen, Zhou Yuyang, Shan Junjie, Zhou Xibin, and Yuan Fajie. Saprot: Protein language modeling with structure-aware vocabulary. BioRxiv, pages 2023–10, 2023.

[43] The UniProt Consortium. Uniprot: the universal protein knowledgebase. Nucleic acids research, 46(5): 2699–2699, 2018. 29425356 10.1093/nar/gky092 PMC5861450

[44] Bhatnagar Aadyot, Jain Sarthak, Beazer Joel, Curran Samuel C, Hoffnagle Alexander M, Ching Kyle S, Martyn Michael, Nayfach Stephen, Ruffolo Jeffrey A, and Madani Ali. Scaling unlocks broader generation and deeper functional understanding of proteins. bioRxiv, pages 2025–04, 2025.

[45] Hsu Chloe, Nisonoff Hunter, Fannjiang Clara, and Listgarten Jennifer. Learning protein fitness models from evolutionary and assay-labeled data. Nature biotechnology, 40(7):1114–1122, 2022. 10.1038/s41587-021-01146-5 35039677

[46] Riesselman Adam J, Ingraham John B, and Marks Debora S. Deep generative models of genetic variation capture the effects of mutations. Nature methods, 15(10):816–822, 2018. 30250057 10.1038/s41592-018-0138-4 PMC6693876

[47] Bradley Ralph Allanand Terry Milton E. Rank analysis of incomplete block designs: I. the method of paired comparisons. Biometrika, 39(3/4):324–345, 1952.

[48] Beltran Antoni, Jiang Xiang’er, Shen Yue, and Lehner Ben. Site-saturation mutagenesis of 500 human protein domains. Nature, 637(8047):885–894, 2025. 39779847 10.1038/s41586-024-08370-4 PMC11754108

[49] Erwood Steven, Bily Teija MI, Lequyer Jason, Yan Joyce, Gulati Nitya, Brewer Reid A, Zhou Liangchi, Pelletier Laurence, Ivakine Evgueni A, and Cohn Ronald D. Saturation variant interpretation using crispr prime editing. Nature Biotechnology, 40(6):885–895, 2022. 10.1038/s41587-021-01201-1 35190686

[50] Starr Tyler N, Greaney Allison J, Hilton Sarah K, Ellis Daniel, Crawford Katharine HD, Dingens Adam S, Navarro Mary Jane, Bowen John E, Tortorici M Alejandra, Walls Alexandra C, Deep mutational scanning of sars-cov-2 receptor binding domain reveals constraints on folding and ace2 binding. cell, 182(5):1295–1310, 2020. 32841599 10.1016/j.cell.2020.08.012 PMC7418704

[51] Muhammad Ayesha, Calandranis Maria E, Li Bian, Yang Tao, Blackwell Daniel J, Harvey M Lorena, Smith Jeremy E, Chew Ashli E, Capra John A, Matreyek Kenneth A, High-throughput functional mapping of variants in an arrhythmia gene. KCNE1, reveals novel biology. bioRxiv, 2028(2023):10–1101, 2023. 10.1186/s13073-024-01340-5 PMC11138074 38816749

[52] McShan Andrew C, Devlin Christine A, Overall Sarah A, Park Jihye, Toor Jugmohit S, Moschidi Danai, Flores-Solis David, Choi Hannah, Tripathi Sarvind, Procko Erik, Molecular determinants of chaperone interactions on mhc-i for folding and antigen repertoire selection. Proceedings of the National Academy of Sciences, 116(51):25602–25613, 2019. 10.1073/pnas.1915562116 PMC6926029 31796585

[53] Chiasson MA, Rollins NJ, Stephany JJ, Sitko KA, Matreyek KA, Verby M, Multiplexed measurement of variant abundance and activity reveals vkor topology, active site and human variant impact. elife [internet]. 2020; 9. Article CAS PubMed PubMed Central. 10.7554/eLife.58026 PMC7462613 32870157

[54] Wu NC, Young AP, Al-Mawsawi LQ, Olson CA, Feng J, Qi H, Chen SH, Lu IH, Lin CY, Chin RG, High-throughput profiling of influenza a virus hemagglutinin gene at single-nucleotide resolution. sci rep 4: 4942, 2014. 24820965 10.1038/srep04942 PMC4018626

[55] Doud Michael B and Bloom Jesse D. Accurate measurement of the effects of all amino-acid mutations on influenza hemagglutinin. Viruses, 8(6):155, 2016. 27271655 10.3390/v8060155 PMC4926175

[56] Roscoe Benjamin P, Thayer Kelly M, Zeldovich Konstantin B, Fushman David, and Bolon Daniel NA. Analyses of the effects of all ubiquitin point mutants on yeast growth rate. Journal of molecular biology, 425(8):1363–1377, 2013. 23376099 10.1016/j.jmb.2013.01.032 PMC3615125

[57] Mavor David, Barlow Kyle, Thompson Samuel, Barad Benjamin A, Bonny Alain R, Cario Clinton L, Gaskins Garrett, Liu Zairan, Deming Laura, Axen Seth D, Determination of ubiquitin fitness landscapes under different chemical stresses in a classroom setting. Elife, 5:e15802, 2016. 10.7554/eLife.15802 PMC4862753 27111525

[58] Roscoe Benjamin Pand Bolon Daniel NA. Systematic exploration of ubiquitin sequence, e1 activation efficiency, and experimental fitness in yeast. Journal of molecular biology, 426(15):2854–2870, 2014. 24862281 10.1016/j.jmb.2014.05.019 PMC4102620

[59] Vanella Rosario, Küng Christoph, Schoepfer Alexandre A, Doffini Vanni, Ren Jin, and Nash Michael A. Understanding activity-stability tradeoffs in biocatalysts by enzyme proximity sequencing. Nature Communications, 15(1):1807, 2024. 10.1038/s41467-024-45630-3 PMC10902396 38418512

[60] Meitlis Iana, Allenspach Eric J, Bauman Bradly M, Phan Isabelle Q, Dabbah Gina, Schmitt Erica G, Camp Nathan D, Torgerson Troy R, Nickerson Deborah A, Bamshad Michael J, Multiplexed functional assessment of genetic variants in card11. The American Journal of Human Genetics, 107(6): 1029–1043, 2020. 33202260 10.1016/j.ajhg.2020.10.015 PMC7820631

[61] Tripathi Arti, Gupta Kritika, Khare Shruti, Jain Pankaj C, Patel Siddharth, Kumar Prasanth, Pulianmackal Ajai J, Aghera Nilesh, and Varadarajan Raghavan. Molecular determinants of mutant phenotypes, inferred from saturation mutagenesis data. Molecular biology and evolution, 33(11):2960–2975, 2016. 27563054 10.1093/molbev/msw182 PMC5062330

[62] Gersing S, Cagiada M, Gebbia M, Gjesing AP, Coté AG, Seesankar G, Li R, Tabet D, Weile J, Stein A, A comprehensive map of human glucokinase variant activity. zenodo. 2023. 10.1186/s13059-023-02935-8 PMC10131484 37101203

[63] Gersing Sarah, Schulze Thea K, Cagiada Matteo, Stein Amelie, Roth Frederick P, Lindorff-Larsen Kresten, and Hartmann-Petersen Rasmus. Characterizing glucokinase variant mechanisms using a multiplexed abundance assay. Genome biology, 25(1):98, 2024. 38627865 10.1186/s13059-024-03238-2 PMC11021015

[64] Coyote-Maestas Willow, Nedrud David, He Yungui, and Schmidt Daniel. Determinants of trafficking, conduction, and disease within a k+ channel revealed through multiparametric deep mutational scanning. Elife, 11:e76903, 2022. 10.7554/eLife.76903 PMC9273215 35639599

[65] Yee Sook Wah, Macdonald Christian, Mitrovic Darko, Zhou Xujia, Koleske Megan L, Yang Jia, Silva Dina Buitrago, Grimes Patrick Rockefeller, Trinidad Donovan, More Swati S, The full spectrum of oct1 (slc22a1) mutations bridges transporter biophysics to drug pharmacogenomics. bioRxiv, 2023.

[66] Deng Zhifeng, Huang Wanzhi, Bakkalbasi Erol, Brown Nicholas G, Adamski Carolyn J, Rice Kacie, Muzny Donna, Gibbs Richard A, and Palzkill Timothy. Deep sequencing of systematic combinatorial libraries reveals β-lactamase sequence constraints at high resolution. Journal of molecular biology, 424 (3–4):150–167, 2012. 23017428 10.1016/j.jmb.2012.09.014 PMC3524589

[67] Firnberg Elad, Labonte Jason W, Gray Jeffrey J, and Ostermeier Marc. A comprehensive, high-resolution map of a gene’s fitness landscape. Molecular biology and evolution, 31(6):1581–1592, 2014. 24567513 10.1093/molbev/msu081 PMC4032126

[68] Jacquier Hervé, Birgy André, Nagard Hervé Le, Mechulam Yves, Schmitt Emmanuelle, Glodt Jérémy, Bercot Beatrice, Petit Emmanuelle, Poulain Julie, Barnaud Guilène, Capturing the mutational landscape of the beta-lactamase tem-1. Proceedings of the National Academy of Sciences, 110(32): 13067–13072, 2013. 10.1073/pnas.1215206110 PMC3740883 23878237

[69] Stiffler Michael A, Hekstra Doeke R, and Ranganathan Rama. Evolvability as a function of purifying selection in tem-1 β-lactamase. Cell, 160(5):882–892, 2015. 25723163 10.1016/j.cell.2015.01.035

[70] Li Xianghua, Lalíc Jasna, Baeza-Centurion Pablo, Dhar Riddhiman, and Lehner Ben. Changes in gene expression predictably shift and switch genetic interactions. Nature communications, 10(1):3886, 2019. 10.1038/s41467-019-11735-3 PMC6715729 31467279

[71] Nguyen Thuy N, Ingle Christine, Thompson Samuel, and Reynolds Kimberly A. The genetic landscape of a metabolic interaction. Nature communications, 15(1):3351, 2024. 10.1038/s41467-024-47671-0 PMC11026382 38637543

[72] Thompson Samuel, Zhang Yang, Ingle Christine, Reynolds Kimberly A, and Kortemme Tanja. Altered expression of a quality control protease in e. coli reshapes the in vivo mutational landscape of a model enzyme. Elife, 9:e53476, 2020. 10.7554/eLife.53476 PMC7377907 32701056

[73] Giacomelli Andrew O, Yang Xiaoping, Lintner Robert E, McFarland James M, Duby Marc, Kim Jaegil, Howard Thomas P, Takeda David Y, Ly Seav Huong, Kim Eejung, Mutational processes shape the landscape of tp53 mutations in human cancer. Nature genetics, 50(10):1381–1387, 2018. 30224644 10.1038/s41588-018-0204-y PMC6168352

[74] Kotler Eran, Shani Odem, Goldfeld Guy, Maya Lotan-Pompan Ohad Tarcic, Gershoni Anat, Hopf Thomas A, Marks Debora S, Oren Moshe, and Segal Eran. A systematic p53 mutation library links differential functional impact to cancer mutation pattern and evolutionary conservation. Molecular cell, 71(1):178–190, 2018. 29979965 10.1016/j.molcel.2018.06.012

[75] Olson C Anders, Wu Nicholas C, and Sun Ren. A comprehensive biophysical description of pairwise epistasis throughout an entire protein domain. Current biology, 24(22):2643–2651, 2014. 25455030 10.1016/j.cub.2014.09.072 PMC4254498

[76] Wu Nicholas C, Dai Lei, Olson C Anders, Lloyd-Smith James O, and Sun Ren. Adaptation in protein fitness landscapes is facilitated by indirect paths. Elife, 5:e16965, 2016. 10.7554/eLife.16965 PMC4985287 27391790

[77] Amorosi Clara J, Chiasson Melissa A, McDonald Matthew G, Wong Lai Hong, Sitko Katherine A, Boyle Gabriel, Kowalski John P, Rettie Allan E, Fowler Douglas M, and Dunham Maitreya J. Massively parallel characterization of cyp2c9 variant enzyme activity and abundance. The American Journal of Human Genetics, 108(9):1735–1751, 2021. 34314704 10.1016/j.ajhg.2021.07.001 PMC8456167

[78] Matreyek Kenneth A, Stephany Jason J, Ahler Ethan, and Fowler Douglas M. Integrating thousands of pten variant activity and abundance measurements reveals variant subgroups and new dominant negatives in cancers. Genome Medicine, 13(1):165, 2021. 34649609 10.1186/s13073-021-00984-x PMC8518224

[79] Mighell Taylor L, Evans-Dutson Sara, and O’Roak Brian J. A saturation mutagenesis approach to understanding pten lipid phosphatase activity and genotype-phenotype relationships. The American Journal of Human Genetics, 102(5):943–955, 2018. 29706350 10.1016/j.ajhg.2018.03.018 PMC5986715

[80] Ahler Ethan, Register Ames C, Chakraborty Sujata, Fang Linglan, Dieter Emily M, Sitko Katherine A, Vidadala Rama Subba Rao, Trevillian Bridget M, Golkowski Martin, Gelman Hannah, A combined approach reveals a regulatory mechanism coupling src’s kinase activity, localization, and phosphotransferase-independent functions. Molecular cell, 74(2):393–408, 2019. 30956043 10.1016/j.molcel.2019.02.003 PMC6474823

[81] Chakraborty Sujata, Ahler Ethan, Simon Jessica J, Fang Linglan, Potter Zachary E, Sitko Katherine A, Stephany Jason J, Guttman Miklos, Fowler Douglas M, and Maly Dustin J. Profiling of the drug resistance of thousands of src tyrosine kinase mutants uncovers a regulatory network that couples autoinhibition to catalytic domain dynamics. BioRxiv, pages 2021–12, 2021. 10.1016/j.chembiol.2023.08.005 PMC10902203 37683649

[82] Nguyen Vanessa, Ahler Ethan, Sitko Katherine A, Stephany Jason J, Maly Dustin J, and Fowler Douglas M. Molecular determinants of hsp90 dependence of src kinase revealed by deep mutational scanning. Protein Science, 32(7):e4656, 2023. 37167432 10.1002/pro.4656 PMC10273359

