# Supplementary material for: SynFit: Synergistic Contrastive Learning for Multi-Objective Protein Fitness Prediction and Optimization
Source: bioRxiv. 2026 May 26:2026.05.21.726972. Preprint. [Version 1] doi: 10.64898/2026.05.21.726972 (PMC13232210; doi:10.64898/2026.05.21.726972)
Supplement: Supplement 1 [file NIHPP2026.05.21.726972v1-supplement-1.pdf]

bioRxiv preprint doi: https://doi.org/10.64898/2026.05.21.726972; this version posted May 26, 2026. The copyright holder for this preprint (which was not certified by peer review) is the author/funder, who has granted bioRxiv a license to display the preprint in perpetuity. It is made available under a CC-BY 4.0 International license.

Supplementary Materials

Supplementary Methods

A.1 Non-dominated Sorting

The complete selection pipeline integrates four complementary algorithms to identify optimal protein mutations from a large candidate space. First, **Algorithm 1** (Non-Dominated Sorting) orchestrates the hierarchical ranking process by repeatedly calling **Algorithm 2** (ExtractNonDominatedSet) to partition solutions into Pareto fronts, where each front represents a distinct level of multi-objective optimality. The domination relationships are determined by **Algorithm 3** (Dominates), which implements the formal definition of Pareto dominance under maximization objectives—ensuring that solutions in earlier fronts are superior trade-offs between competing objectives. Finally, **Algorithm 4** (SelectTopK) addresses the practical constraint of selecting a fixed number of variants for experimental validation by including complete high-quality fronts and applying an average-score tie-breaking criterion on the boundary front. This ensures the final selection maintains both Pareto optimality (by prioritizing better fronts) and internal quality (by selecting the best candidates within the partial front), producing a diverse yet high-performing set of mutations that span the Pareto frontier while respecting experimental budget constraints. In practice, we utilized the PyGMO library's fast non-dominated sorting implementation, which employs efficient data structures to achieve $O(MN^2)$ complexity for the complete sorting process.

Algorithm 1 Non-Dominated Sorting for Multi-Objective Optimization. This algorithm partitions a population of solutions into hierarchical Pareto fronts based on dominance relationships. It iteratively extracts non-dominated solutions to form each front, where Front 1 contains globally optimal solutions (the Pareto frontier), Front 2 contains solutions dominated only by Front 1, until all solutions are assigned a rank.

Require: Set of solutions $P = \{p_1, p_2, \ldots, p_n\}$, where each p_i has objective values $(f_1(p_i), f_2(p_i), \ldots, f_m(p_i))$
Ensure: List of Pareto fronts $\mathcal{F} = \{F_1, F_2, \ldots, F_k\}$

1: $\mathcal{F} \leftarrow \emptyset$ ▷ Initialize list of fronts
2: $P_{\text{remaining}} \leftarrow P$ ▷ Pool of unassigned solutions
3: rank $\leftarrow 1$
4: **while** $P_{\text{remaining}} \neq \emptyset$ **do**
5:     $F_{\text{rank}} \leftarrow$ ExtractNonDominatedSet($P_{\text{remaining}}$)
6:     $\mathcal{F} \leftarrow \mathcal{F} \cup \{F_{\text{rank}}\}$
7:     $P_{\text{remaining}} \leftarrow P_{\text{remaining}} \setminus F_{\text{rank}}$
8:     rank $\leftarrow$ rank $+ 1$
9: **end while**
10: **return** $\mathcal{F}$

bioRxiv preprint doi: https://doi.org/10.64898/2026.05.21.726972; this version posted May 26, 2026. The copyright holder for this preprint (which was not certified by peer review) is the author/funder, who has granted bioRxiv a license to display the preprint in perpetuity. It is made available under a CC-BY 4.0 International license.

Algorithm 2 ExtractNonDominatedSet. This subroutine identifies the set of non-dominated solutions. For each candidate solution, it checks whether any other solution dominates it; if no dominating solution exists, the candidate is added to the non-dominated set. This forms the basis for constructing each Pareto front in the sorting process.

```
Require: Set of solutions P
Ensure: Non-dominated set F ⊆ P
F ← ∅                                          ▷ Non-dominated set
for each solution p_i ∈ P do
    is_dominated ← False
    for each solution p_j ∈ P where i ≠ j do
        if DOMINATES(p_j, p_i) then
            is_dominated ← True
            break                              ▷ No need to check further
        end if
    end for
    if ¬is_dominated then
        F ← F ∪ {p_i}
    end if
end for
return F
```

Algorithm 3 Dominates (Maximization). This function determines whether solution p_i dominates solution p_j under a maximization objective framework. Dominance occurs when p_i is at least as good as p_j in all objectives and strictly better in at least one objective. The function returns true only when both conditions are satisfied, enabling proper Pareto frontier identification.

```
Require: Solutions p_i and p_j with m objectives
Ensure: Boolean indicating if p_i dominates p_j
better_in_one ← False
at_least_as_good_in_all ← True
for each objective k = 1 to m do
    if f_k(p_i) > f_k(p_j) then
        better_in_one ← True                   ▷ Strictly better in at least one
    else if f_k(p_i) < f_k(p_j) then
        at_least_as_good_in_all ← False        ▷ Worse in some objective
        break
    end if
end for
return better_in_one ∧ at_least_as_good_in_all
```

bioRxiv preprint doi: https://doi.org/10.64898/2026.05.21.726972; this version posted May 26, 2026. The copyright holder for this preprint (which was not certified by peer review) is the author/funder, who has granted bioRxiv a license to display the preprint in perpetuity. It is made available under a CC-BY 4.0 International license.

Algorithm 4 SelectTopK with Tie-Breaking. This algorithm selects exactly K solutions from the ranked Pareto fronts for experimental validation. It greedily includes complete fronts sequentially until adding the next front would exceed K. For the final partial front, it breaks ties by ranking solutions based on their average objective score and selecting the top-ranked solutions to reach exactly K total, ensuring both quality and diversity in the final selection.

Require: List of Pareto fronts $\mathcal{F} = \{F_1, F_2, \ldots, F_k\}$, target count K
Ensure: Set of top K solutions S

$S \leftarrow \emptyset$ ▷ Selected solutions
rank $\leftarrow 1$
while rank $\leq |\mathcal{F}|$ **do**
  if $|S| + |F_{\text{rank}}| \leq K$ **then**
    $S \leftarrow S \cup F_{\text{rank}}$ ▷ Include entire front
    rank $\leftarrow$ rank $+ 1$
  else
    break ▷ Next front would exceed limit
  end if
end while
if $|S| < K$ **then** ▷ Need to select from next front
  $n_{\text{needed}} \leftarrow K - |S|$
  $F_{\text{partial}} \leftarrow F_{\text{rank}}$ ▷ The front to break ties on
  for each solution $p \in F_{\text{partial}}$ **do**
    p.avg_score $\leftarrow \frac{1}{m} \sum_{k=1}^{m} f_k(p)$ ▷ Compute average score
  end for
  Sort F_{partial} by avg_score in descending order
  $S \leftarrow S \cup F_{\text{partial}}[1 : n_{\text{needed}}]$ ▷ Select top n_{needed} solutions
end if
return S

bioRxiv preprint doi: https://doi.org/10.64898/2026.05.21.726972; this version posted May 26, 2026. The copyright holder for this preprint (which was not certified by peer review) is the author/funder, who has granted bioRxiv a license to display the preprint in perpetuity. It is made available under a CC-BY 4.0 International license.

B Supplementary Tables

B.1 Multi-fitness benchmark datasets

We initially collected 22 proteins encompassing 50 deep mutational scanning (DMS) datasets from ProteinGym[16], focusing on proteins for which multiple functional assays were available. To ensure sufficient coverage and computational feasibility, we excluded the NPC1_HUMAN dataset[49], which contained only 58 variants and was too small for reliable benchmarking, and omitted SPIKE_SARS2[50] due to its long sequence length exceeding GPU memory limits during model training. After filtering, our final benchmark comprised 20 proteins with 46 functional assays spanning diverse reaction types and molecular functions, forming a large-scale multi-fitness dataset for evaluating cross-objective generalization in SynFit. In section 2.3, due to the fact that some DMS assays measure overlapping functions or lack wild-type references, our analysis was limited to eight proteins – *CP2C9_HUMAN*, *HXK4_HUMAN*, *KCNE1_HUMAN*, *RL40A_YEAST*, *KCNJ2_MOUSE*, *OXDA_RHOTO*, *PTEN_HUMAN*, and *RASK_HUMAN* – spanning 16 assays in total.

**UniProt ID**	**#Variants**	**Fitness Type(s)**
SPIKE_SARS2	3,798	Binding[50], Expression[50]
KCNE1_HUMAN	2,313	Expression[51], Activity[51]
Q53Z42_HUMAN	3,345	Binding[52], Expression[52]
VKOR1_HUMAN	693	Expression[53], Activity[53]
A0A2Z5U3Z0_9INFA	2,351	Growth Rate[54], Growth Rate[55]
RL40A_YEAST	1,139	Growth Rate[56], Growth Rate[57], Activity[58]
OXDA_RHOTO	6,388	Activity[59], Expression[59]
CAR11_HUMAN	2,355	Growth Rate[60], Growth Rate[60]
CCDB_ECOLI	1,150	Activity[61], Growth Rate[61]
HXK4_HUMAN	8,256	Growth Rate[62], Expression[63]
KCNJ2_MOUSE	6,790	Activity[64], Expression[64]
S22A1_HUMAN	9,716	Expression[65], Activity[65]
BLAT_ECOLX	989	Growth Rate[66], Growth Rate[67], Growth Rate[68], Growth Rate[69]
RPC1_LAMBD	352	Activity[70], Activity[70]
DYR_ECOLI	2,362	Growth Rate[71], Growth Rate[72]
P53_HUMAN	1,049	Growth Rate[73], Growth Rate[73], Growth Rate[73], Growth Rate[74]
RASK_HUMAN	23,073	Expression[33], Binding[33]
SPG1_STRSG	1,459	Binding[75], Binding[76]
CP2C9_HUMAN	4,422	Expression[77], Activity[77]
PTEN_HUMAN	4,840	Expression[78], Activity[79]
SRC_HUMAN	3,357	Activity[80], Activity[81], Growth Rate[82]

Table S1: Summary of the multi-fitness benchmark curated from ProteinGym, containing 46 DMS assays across 20 proteins with diverse functional measurements such as binding, expression, and growth rates, supporting comprehensive evaluation of SynFit models.

bioRxiv preprint doi: https://doi.org/10.64898/2026.05.21.726972; this version posted May 26, 2026. The copyright holder for this preprint (which was not certified by peer review) is the author/funder, who has granted bioRxiv a license to display the preprint in perpetuity. It is made available under a CC-BY 4.0 International license.

C Supplementary Figures

C.1 Complete multi-objective pareto analysis results

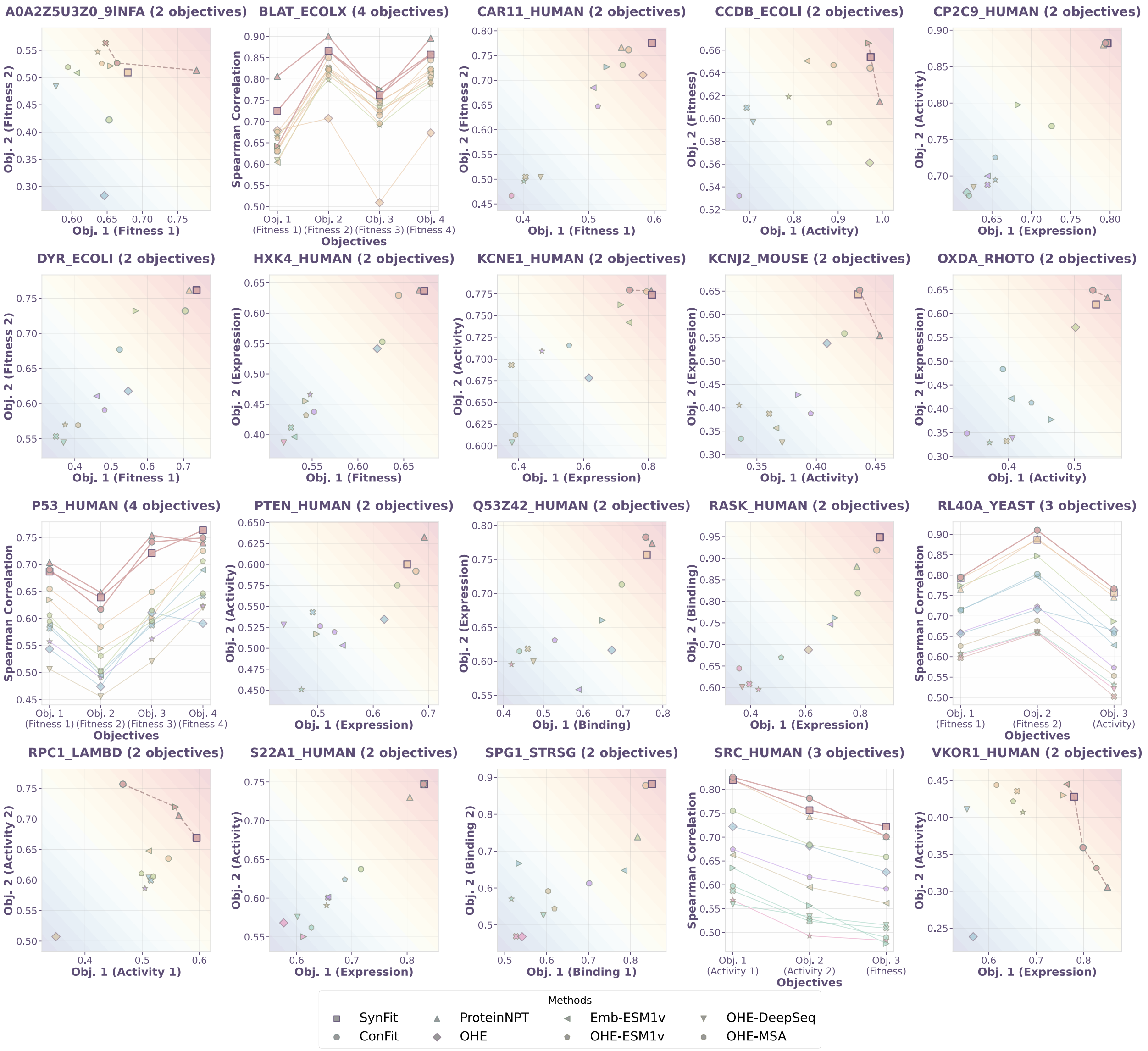

Figure S1: Multi-objective protein Pareto analysis. Comprehensive Pareto analysis across the first subset of proteins in the multi-fitness dataset. Each panel summarizes trade-offs between distinct fitness objectives (e.g., activity, stability, expression, binding) for SynFit and baseline models, with Pareto fronts highlighting the advantage of multi-objective joint training.

bioRxiv preprint doi: https://doi.org/10.64898/2026.05.21.726972; this version posted May 26, 2026. The copyright holder for this preprint (which was not certified by peer review) is the author/funder, who has granted bioRxiv a license to display the preprint in perpetuity. It is made available under a CC-BY 4.0 International license.

C.2 Functional Site Identification

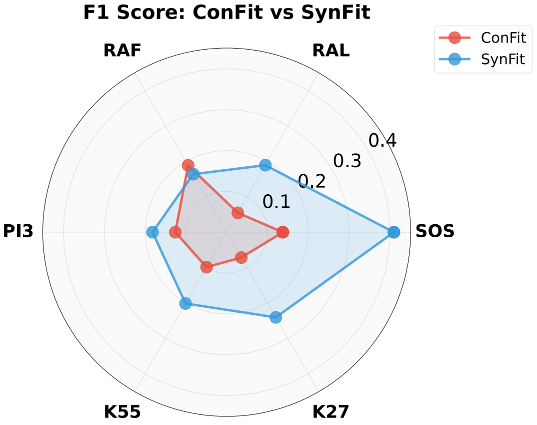

(a) F1 comparison (ConFit vs. SynFit).

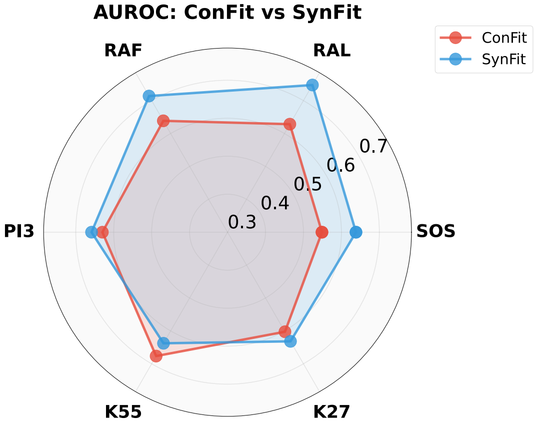

(b) AUROC comparison (ConFit vs. SynFit).

Figure S2: Function site identification SynFit vs. ConFit AUROC and F1 score performance Side-by-side comparison of F1 and AUROC across six KRAS binding partners. SynFit consistently outperforms ConFit on both metrics on five out of the binders for functional site identification, indicating improved classification performance.
